# Glymphatic System Visualized With Intrathecal Gadoterate Meglumine Administered via External Ventricular Drain

**DOI:** 10.7759/cureus.107881

**Published:** 2026-04-28

**Authors:** Christopher Wong, Imran Siddiqi, Louis Reier, Alice Wang, Maxwell Marino, Konstantin Tchalukov, Christopher Nguyen, Dan E Miulli

**Affiliations:** 1 Neurological Surgery, Riverside University Health System Medical Center, Moreno Valley, USA; 2 Neurological Surgery, Arrowhead Regional Medical Center, Colton, USA; 3 Radiology, Riverside University Health System Medical Center, Moreno Valley, USA

**Keywords:** external ventricular drain, glymphatic, neurosurgery, osteopathic, traumatic brain injury

## Abstract

Background

Traumatic brain injury (TBI) and other acute intracranial pathologies disrupt the glymphatic system, a recently described waste-clearance network that facilitates the removal of metabolic byproducts from the brain. Dysfunction of this system after injury may contribute to impaired clearance of toxic metabolites, cerebral edema, and elevated intracranial pressure. This study aimed to evaluate glymphatic dynamics using intrathecal (IT) administration of gadoterate meglumine via an external ventricular drain (EVD) to better understand the impact of intracranial injury on glymphatic flow.

Methods

This single-center retrospective study, conducted from July 2025 to November 2025, enrolled six patients who were admitted for an intracranial pathology that required placement of an EVD to study the glymphatic system utilizing IT administration of contrast agent gadoterate meglumine. Serial magnetic resonance imaging (MRI) brain scans were performed pre-contrast and at least one additional time, either four, 12, or 36 hours after contrast administration. Intensity measurements were then taken on the images and compared at the following brain parenchymal locations: bilateral frontal white and grey matter, bilateral temporal white and grey matter, bilateral parietal white and grey matter, central pons located ventral to the cerebral aqueduct, central medulla located ventral to the fourth ventricle, and bilateral cerebellar white matter.

Results

No immediate procedural complications were observed following IT contrast administration. Patients demonstrated variable degrees of MRI signal change that correlated with presumed glymphatic function, with increased enhancement observed in regions of preserved flow and reduced enhancement in areas affected by intracranial pathology. In some cases, transient decreases in signal intensity were observed following contrast administration, which may reflect localized high-concentration contrast effects with susceptibility-related signal loss (“first-pass” phenomenon), altered glymphatic transport, or impaired clearance. Reduced glymphatic tracer propagation and diminished enhancement were observed in patients with neurological decline, whereas enhanced glymphatic transport was noted following EVD placement. One patient experienced neurological deterioration following IT contrast administration; however, given the presence of multiple confounding clinical factors, a causal relationship could not be established.

Conclusions

The glymphatic system plays a critical role in intracranial homeostasis and appears to be disrupted following acute brain injury. IT contrast-enhanced MRI enables visualization of glymphatic dynamics and demonstrates regional variation in tracer movement corresponding to underlying pathology. While low-dose IT gadolinium administration has demonstrated tolerability in prior human studies, the present findings highlight that definitive conclusions regarding safety and causality cannot be drawn from this small cohort. These results support the feasibility of IT contrast-enhanced MRI for evaluating glymphatic function, but larger prospective studies are needed to better define safety, pathophysiological mechanisms, and clinical implications.

## Introduction

The glymphatic system clears the brain interstitial space and brain cellular solutes and maintains brain tissue homeostasis [1]. Cerebrospinal fluid (CSF) from the subarachnoid space travels along the pathways of penetrating arteries and arterioles as they enter the brain parenchyma. With pulsations, CSF moves into the perivascular Virchow-Robin spaces (VRS). These perivascular extensions of the pia mater surround the perforating arteries and veins along the lenticulostriate arteries and medullary perforating arteries in the basal ganglia and cerebral peduncles and create a space that enlarges with age. Flow of CSF into VRS decreases due to factors that disrupt the normal pulsatile "perivascular pump" that drives CSF movement, such as arteriosclerosis seen in cerebrovascular disease. Over 40% of subarachnoid CSF enters the brain parenchyma alongside the VRS perivascular spaces, becoming glymphatic fluid. The walls of these perivascular spaces are lined by gatekeepers called astrocyte foot processes, which are densely packed with Aquaporin-4 (AQP-4) water channels and facilitate the exchange of CSF with interstitial fluid (ISF) in the glymphatic system. These AQP-4 channels allow for rapid, facilitated movement of water and solutes from the CSF into the brain parenchyma’s spaces and cells through the glymphatic system, exiting the brain substance, collecting into cisterns, and passing into the lymphatic system. CSF surrounds and protects the brain and spinal cord, while the ISF surrounds and protects brain tissues and cells. Fluid within each space, whether CSF or the glymphatic system, has a different chemical composition. The CSF influx, driven by arterial pulsation and other physiological factors, creates a convective bulk flow of glymphatic fluid through brain parenchyma that then enters the perivenous space to merge with the efflux of fluid, clearing waste products through meningeal lymphatic vessels into cervical lymph nodes [2].

AQP channels, of which there are at least seven in the mammalian central nervous system (CNS), function as bidirectional water-selective channels. There is a more nuanced arrangement of fluid flow contingent not only on the expression of AQP-4, but also the differential expression of a variety of AQP channels, state of the glial tissue, and neurohumoral upregulation and/or downregulation [1,3]. 

Impairment of AQP-4 channel function has been implicated in neurological diseases, including intracranial hemorrhage, ischemic stroke, traumatic brain injury, Alzheimer’s disease, Parkinson’s disease, vascular dementia, idiopathic normal pressure hydrocephalus, and migraines [1]. In pathologies where there is increased cerebral edema, such as brain tumors, intracranial hemorrhage, ischemic stroke, and traumatic brain injury, there is an increase in AQP-4 channels through upregulation of mRNA expression, suggesting the need to improve flow to clear up the accumulation of fluid, metabolites, and byproducts of primary and secondary brain injury [4]. In patients with Alzheimer’s disease, high levels of norepinephrine or increased progesterone are associated with a decrease in AQP-4 channels, which further impairs the clearance of solutes, such as amyloid-beta and tau proteins in Alzheimer’s disease via the glymphatic system.

Both physiological mechanisms and specific pharmacological agents can increase AQP-4 brain water permeability within the glymphatic system [1,3]. Hypotonic conditions around astrocytes mediated by calcium/calmodulin and protein kinase signaling can trigger rapid fluid movement. Other conditions that increase AQP-4 channel activity include vasopressin-related protein synthesis, upregulation of some AQP channels, lead, manganese, and cysteine leukotriene receptor activation. These conditions result in increased fluid into the brain parenchyma, stimulating glymphatic system activity. However, fluid and metabolites must travel through the glymphatic system in order not to be pathogenic. The glymphatic system clears out these substances to the perivenous spaces and takes the debris with the convective flow. This is enhanced with arterial pulsations, pressure gradients within the brain parenchyma, osmotic forces, increased neuronal activity particularly around synapses and with astrocyte motility, voluntary exercises leading to AQP-4 polarization, increased clearance of glymphatic fluid during exhaling respiration when there is a pulling or suctioning effect out of the brain parenchyma into the perivenous spaces for drainage, and during deepest non-REM sleep with a predominance of delta waves 0.5-4 Hz when the brain interstitial space expands by possibly 60% reducing the resistance to bulk flow.

Intravenous (IV) injection of gadolinium, such as when given to enhance MRI imaging, travels through a disrupted blood-brain barrier and accumulates in the brain interstitial space, drawing fluids from nearby cells. It may also travel through the perivascular system and the glymphatic system into the brain parenchyma cells through fluid leakage. Intrathecal (IT) injection of gadolinium-based contrast agents (GBCAs) has been used to study the glymphatic pathway. After mixing with CSF, the contrast moves into the brain parenchyma through the perivascular spaces and AQP-4 channels through arterial pulsations, usually over four to 12 hours. IT lumbar puncture administration of the MRI GBCAs gadobutrol (Gadavist, Bayer AG, Leverkusen, Germany), 0.5 ml of 1.0 mmol/ml serving as a CSF tracer, showed glymphatic drainage in the human brain (inferior frontal gyrus, pons, thalamus, parahippocampal gyrus, and adjacent CSF) to cervical lymph nodes [5]. In the study of 19 patients, enhancement of the CSF was observed before glymphatic enhancement. Brain glymphatic enhancement is followed by cervical lymph node enhancement, peaking after 24 hours rather than four to six hours for peaked glymphatic enhancement. In a cohort of 30 patients with idiopathic normal pressure hydrocephalus (iNPH) compared to eight patients with suspected CSF leak, patients with iNPH have delayed clearance (persistent presence of contrast over time) of IT gadobutrol (Gadavist) via lumbar puncture from the CSF, entorhinal cortex, and adjacent white matter, suggesting a dysfunctional glymphatic system [6]. Gadobutrol clearance from the CSF is delayed at 24 and 48 hours post-IT lumbar injection [7].

Prior studies using IT GBCAs were off-label use because they are not FDA approved for IT diagnostic purposes, and their safety profile has been described. Different GBCAs have different safety profiles. IT gadobenate dimeglumine (MultiHance, Bracco S.p.A., Milan, Italy) injection full prescribing information contains the warning that it can cause serious adverse events, including seizures, encephalopathy, coma, and death. IV gadobenate dimeglumine (MultiHance) can increase the risk of nephrogenic systemic fibrosis in patients with acute kidney injury or chronic, severe kidney disease. In a cohort of 100 patients receiving IT gadobutrol (Gadavist) and iodixanol via lumbar puncture for iNPH, adverse events included anaphylaxis in one patient with a known allergic reaction to iodine-containing contrast agent, temporary severe headache (28%), and severe nausea (34%), which was thought to be due to the iNPH diagnosis [8]. The mean time from IT administration of gadobutrol via lumbar puncture until the first visual detection at the foramen magnum was 20 ± 23 minutes. In a systematic review and meta-analysis, IT doses less than 1.0 mmol were deemed safe with fewer neurotoxic side effects [9]. In a more recent study, 0.1 mmol was found to be too low a dosage to provide diagnostic information, while 0.25 mmol was enough to provide adequate diagnostic information [10].

IV Gadoxetate disodium (Eovist, Bayer AG, Leverkusen, Germany) is FDA-approved for detecting hepatic lesions on MRI, while IV gadoterate meglumine (Dotarem, Guerbet, Villepinte, France) has been FDA-approved for visualization of disruption of the blood-brain barrier (BBB) and abnormal vascularity in the brain, spine, and associated tissues in both pediatric and adult patients. However, there is no clear delineation of the CSF glymphatic pathways through which the contrast flows, which this study was designed to investigate. To date, there have been no studies demonstrating IT use of either gadoxetate disodium (Eovist) or gadoterate meglumine (Dotarem). The safety of the IT administration of these two GBCAs is unknown. However, a recent study investigated the safety of IV gadoterate meglumine over 35 years of clinical use with more than 170 million doses given and found minimal adverse events (urticaria (0.001%), nausea (0.001%), vomiting (0.001%), and hypersensitivity reactions (< 0.00035%) [11]. Given these findings, gadoterate meglumine (Dotarem) was chosen as the IT GBCA via external ventricular drain (EVD) for the study.

IT delivery is invasive and requires either a lumbar puncture or placement of an EVD. EVD placement is a neurosurgical procedure that serves as a diagnostic and therapeutic intervention [12]. Common indications for placing an EVD include hydrocephalus from intracranial hemorrhage or tumors and close neurological monitoring in traumatic brain injury patients with depressed Glasgow Coma Scale (GCS) scores [13].

The safety profile of IT GBCAs is not yet well understood, and tracing the glymphatic pathway is time-dependent and costly because it requires serial MRI images. While previous studies delivered IT GBCA via lumbar puncture, IT GBCA has also been given via an EVD. Two case reports described accidental administration of GBCA via the IT route through an EVD instead of IV due to misinterpretation of the lines [14,15]. Both patients suffered irreversible neurological damage. The dose for IV administration was higher (more than 1.0 mmol) than those studied when given the IT route via lumbar puncture, and prior studies have shown severe neurotoxicity associated with high doses of IT GBCA [9]. When the IT GBCA is given at a lower dose, the side effects should be similar regardless of the administration via lumbar puncture or EVD route, including headache, nausea, and dizziness [8]. If IT GBCA can be safely administered via EVD, this would allow for widespread study of the glymphatic system in patients with intracranial pathologies that require EVD placement, including traumatic brain injury and iNPH [3].

MRI scans of the brain are used to characterize the brain parenchyma and evaluate a multitude of pathologies, including tumors, cerebral edema, ischemia, and hemorrhage. Methods to standardize the intensity values utilized in MRI measurements have been improving since the creation of the MRI machine; however, there is no universally accepted meaning of the intensity unit [16]. Therefore, there is no standard process to perform quantitative analysis of MRI image intensity values across multiple institutions. However, prior studies have shown that the intensity unit is standardized between different MRI scans if they are performed on the same machine with the same viewing parameters set up for each run [17].

Here, we describe several case reports of IT administration of gadoterate meglumine (Dotarem) via an EVD to visualize the CSF glymphatic pathway and characterize glymphatic drainage by measuring the MRI intensity of different brain tissues and spaces.

## Materials and methods

This single-center prospective study, conducted from July 2025 to November 2025, received Institutional Review Board approval (Arrowhead Regional Medical Center; protocol #24-10). Inclusion criteria included patients at least 18 years old and patients who were admitted for an intracranial pathology that required placement of an EVD. Exclusion criteria included patients less than 18 years old, patients with a known allergy to MRI contrast, and those who did not consent to the process. Electronic medical records were reviewed to collect information, including patient characteristics, radiographic findings, treatments, and clinical outcomes. Clinical information was subsequently de-identified.

Six patients were enrolled in the research study. After obtaining consent, IT gadoterate meglumine (Dotarem) (0.5 mmol of 1.0 mmol/ml) was delivered to each patient via the EVD. The contrast serves as a CSF glymphatic fluid tracer and should increase the intensity on MRI images as it exits the ventricular system and flows through the brain parenchyma, exiting and eventually reaching the lymph nodes. If there was an intracranial process that obstructed the glymphatic inflow or outflow, such as hemorrhage, edema, or tumor, we anticipated there may be no change in intensity when compared to the non-obstructed portion of the brain. However, it was unclear if the change in intensity would be affected by different kinds of intracranial pathology and the timing of the MRI scan.

The first patient was enrolled to determine if IT contrast administration via EVD was taken up into the brain parenchyma with no adverse events. In the first patient, an MRI brain scan was obtained 12 hours after contrast administration, and it was confirmed that contrast was present in the ventricles and brain parenchyma. For the other five patients, pre-contrast MRI brain scans were obtained in addition to at least one additional MRI brain scan four, 12, or 36 hours after IT contrast administration to determine the timing of contrast uptake within the glymphatic system. All MRI scans were performed on the same MRI machine using the same settings. All completed images were formatted to the exact same window level. Intensity measurements were then taken on the images at each timepoint and compared at the following brain parenchymal locations: left frontal white matter, left frontal grey matter, right frontal white matter, right frontal grey matter, left temporal white matter, left temporal grey matter, right temporal white matter, right temporal grey matter, left parietal white matter, left parietal gray matter, right parietal white matter, right parietal grey matter, central pons located ventral to the cerebral aqueduct, central medulla located ventral to the fourth ventricle, left cerebellar white matter, and right cerebellar white matter.

To standardize the intensity measurements across all the patients, we adjusted the images to have the same window width (W) 5800 and window length (L) 3400. We were unable to adjust the repetition time (TR) and echo time (TE) for each MRI scan because they were predefined at the time of each MRI scan. The TE remained the same at 10. The TR varied from 504 to 609. Differences in MRI intensities between the timepoints at each location were also calculated for the white and grey matter separately. Additionally, the averages of the differences were also calculated.

## Results

Patient 1

A 61-year-old female patient presented with a chief complaint of altered mental status with an initial GCS4T (M2VTE1). A head computed tomography (CT) scan demonstrated right temporal intraparenchymal hemorrhage with diffuse subarachnoid hemorrhage (SAH) and signs of hydrocephalus. Head CT angiography (CTA) scan demonstrated a ruptured four-millimeter aneurysm arising from the right posterior communicating artery. A left-sided EVD was placed for CSF diversion. A diagnostic cerebral angiogram or digital subtraction angiography (DSA) was subsequently performed, and the patient underwent successful coil embolization of the ruptured aneurysm.

An MRI brain scan was obtained 12 hours post-IT contrast with a GCS score of 4T (M2VTE1) at that time point to ensure the contrast could be visualized within the ventricles and brain parenchyma. IT gadoterate meglumine (Dotarem) could be seen in the ventricles and brain parenchyma. The MRI brain scan was performed 36 hours after the CTA and 22 hours after the DSA.

Tables 1-2 display the MRI intensities of patient 1's brain parenchymal regions and lymph nodes 12 hours post-IT contrast to determine if IT contrast is taken up into brain tissue compared to other patients, who averaged an intensity of 1500 in the supratentorial compartment.

**Table 1:** Patient 1 brain parenchyma MRI intensities 12 hours post-IT contrast (TE 10 and TR 504) IT: Intrathecal; TE: Echo time; TR: Repetition time

Location	12 hours post-IT contrast intensity
Left frontal lobe (white matter)	2292
Left frontal lobe (grey matter)	1858
Right frontal lobe (white matter)	2275
Right frontal lobe (grey matter)	1982
Left temporal lobe (white matter)	2841
Left temporal lobe (grey matter)	2359
Right temporal lobe (white matter)	2655
Right temporal lobe (grey matter)	2168
Left parietal lobe (white matter)	2564
Left parietal lobe (grey matter)	2266
Right parietal lobe (white matter)	2675
Right parietal lobe (grey matter)	2202
Central pons (ventral to cerebral aqueduct)	2906
Central medulla (ventral to fourth ventricle)	2527
Left cerebellum (white matter)	2832
Right cerebellum (white matter)	2874

**Table 2:** Patient 1 lymph node MRI intensities 12 hours post-IT contrast (TE 10 and TR 507) IT: Intrathecal; TE: Echo time; TR: Repetition time

Location	12 hours post-IT contrast intensity
Left retropharyngeal	1631
Right retropharyngeal	1465
Left posterior cervical	1345
Right posterior cervical	859

**Figure 1:**
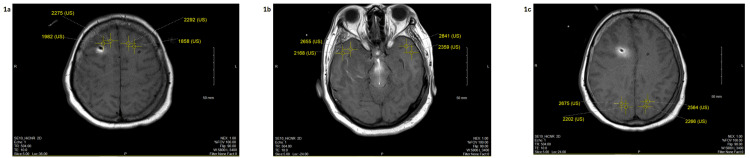
Patient 1 MRI intensities 12 hours post-IT contrast 1a: Frontal lobe; 1b: Temporal lobe; 1c: Parietal lobe IT: Intrathecal

Figure 1 illustrates the MRI intensities measured at each location.

Patient 2

A 51-year-old male patient presented with a chief complaint of altered mental status with an initial GCS4T (M2VTE1). The patient had a left temporal intraparenchymal hemorrhage (IPH) with intraventricular hemorrhage and signs of hydrocephalus. A right-sided EVD was placed for CSF diversion. A DSA was subsequently performed and demonstrated a left dural-based arteriovenous fistula (AVF) with arterial feeders from the left inferior temporal branch of the left internal carotid artery and left meningohypophyseal branch and draining veins into the left vein of Trolard and left transverse venous sinus. The patient then underwent another DSA for Onyx (Medtronic, Dublin, Ireland) embolization of the AVF, followed by a left craniotomy for resection of the lesion.

MRI brain scans were obtained pre-IT contrast and 12 hours post-IT contrast with corresponding GCS scores of 7T (M5VTE1) and 10T (M5VTE4) at those respective timepoints. The pre-IT contrast MRI brain scan was performed 26 hours after the CTA and before the DSA and craniotomy. The post-IT contrast MRI brain scan was performed 297 hours after the CTA, 240 hours after the first DSA, 152 hours after the second DSA, and 129 hours after the craniotomy.

Tables 3-4 display the MRI intensities of patient 2's brain parenchymal regions and lymph nodes pre- and 12 hours post-IT contrast.

**Table 3:** Patient 2 brain parenchyma MRI intensities pre- and 12 hours post-IT contrast IT: Intrathecal; TR: Repetition time

Location	Pre-IT contrast intensity (TR 546)	12 hours post-IT contrast intensity (TR 609)	Change in intensity	% change
Left frontal lobe (white matter)	1521	1713	192	11.6
Left frontal lobe (grey matter)	1170	1468	298	25.5
Right frontal lobe (white matter)	1454	1643	189	13.0
Right frontal lobe (grey matter)	1072	1539	467	43.6
Left temporal lobe (white matter)	1840	1911	71	3.9
Left temporal lobe (grey matter)	1532	1735	203	13.3
Right temporal lobe (white matter)	1716	1975	259	15.1
Right temporal lobe (grey matter)	1585	1658	73	4.6
Left parietal lobe (white matter)	1597	1692	95	5.9
Left parietal lobe (grey matter)	1309	1508	199	15.2
Right parietal lobe (white matter)	1581	1725	144	9.1
Right parietal lobe (grey matter)	1279	1565	286	22.4
Central pons (ventral to cerebral aqueduct)	1641	2476	835	50.9
Central medulla (ventral to fourth ventricle)	1663	1947	284	17.1
Left cerebellum (white matter)	1589	1911	322	20.3
Right cerebellum (white matter)	1866	2301	435	23.3

**Table 4:** Patient 2 lymph node MRI intensities pre- and 12 hours post-IT contrast IT: Intrathecal; TR: Repetition time

Location	Pre-IT contrast intensity (TR 546)	12 hours post-IT contrast intensity (TR 609)	Change in intensity	% change
Left retropharyngeal	2941	2948	7	0.2
Right retropharyngeal	2604	2740	136	5.2
Left posterior cervical	1195	1148	-47	-3.9
Right posterior cervical	1259	1448	189	15.0

Table 5 displays the average change in MRI intensities.

**Table 5:** Patient 2 average change in MRI intensities pre- and 12 hours post-IT contrast IT: Intrathecal

Location	Average change in intensity
White matter	158.3
Grey matter	254.3
Left white matter	119.3
Left grey matter	233.3
Right white matter	197.3
Right grey matter	275.3
Left hemisphere	176.3
Right hemisphere	236.3

**Figure 2:**
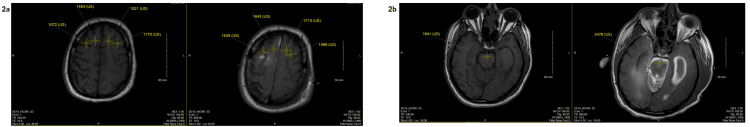
Patient 2 MRI intensities pre- and 12 hours post-IT contrast In each pair of images, the left image is pre-IT contrast, and the right image is 12 hours post-IT contrast. 2a: Frontal lobe; 2b: Pons IT: Intrathecal

Figure 2 illustrates the MRI intensities measured at each location.

There was a higher average increase in intensity in grey matter when compared to white matter. Furthermore, there was also a higher average increase in intensity in the right side of the brain, with the EVD, and opposite the IPH, when compared to the left side of the brain. This may be explained by the patient’s hemorrhage in the left temporal lobe, causing glymphatic obstruction on the left side and higher relative intracranial pressure (ICP), which resulted in less IT contrast uptake and a smaller change in intensity. The EVD, in addition to draining CSF, may have facilitated increasing the drainage of ISF and a better functioning glymphatic pathway.

There was also an increase in intensity in the right-sided retropharyngeal and posterior cervical lymph nodes, which could be explained by the left-sided obstruction due to the IPH.

Patient 3

A 40-year-old male patient presented with a chief complaint of headaches with an initial GCS15 (M6V5E4). The patient had a ventriculoperitoneal shunt (VPS) placed at birth for spina bifida and a history of orphaned VPS catheters. He presented to the hospital with evidence of the distal VPS catheter eroding through his colon. The VPS was removed, and a follow-up head CT scan demonstrated signs of evolving hydrocephalus. A right-sided EVD was placed for CSF diversion.

MRI brain scans were obtained pre-IT contrast and four hours post-IT contrast with GCS scores of 15 (M6V5E4) at both timepoints. There was no MRI scan of the neck. Table 6 displays the MRI intensities of patient 3's brain parenchymal regions pre- and 12 hours post-IT contrast.

**Table 6:** Patient 3 brain parenchyma MRI intensities pre- and four hours post-IT contrast IT: Intrathecal; TR: Repetition time

Location	Pre-IT contrast intensity (TR 504)	Four hours post-IT contrast intensity (TR 504)	Change in intensity	% change
Left frontal lobe (white matter)	2163	2304	141	6.5
Left frontal lobe (grey matter)	1688	2054	366	21.7
Right frontal lobe (white matter)	2084	2120	36	1.7
Right frontal lobe (grey matter)	1620	1858	238	14.7
Left temporal lobe (white matter)	2085	2339	254	12.2
Left temporal lobe (grey matter)	1885	2286	401	21.3
Right temporal lobe (white matter)	2115	2376	261	12.3
Right temporal lobe (grey matter)	1846	2157	311	16.8
Left parietal lobe (white matter)	2035	1931	-104	-5.1
Left parietal lobe (grey matter)	1880	1921	41	2.2
Right parietal lobe (white matter)	1969	1970	1	0.1
Right parietal lobe (grey matter)	1803	1793	-10	-0.6
Central pons (ventral to cerebral aqueduct)	1893	1982	89	4.7
Central medulla (ventral to fourth ventricle)	2069	2060	-9	-0.4
Left cerebellum (white matter)	1961	2490	529	27.0
Right cerebellum (white matter)	2278	2231	-47	-2.1

Table 7 displays the average change in MRI intensities.

**Table 7:** Patient 3 average change in MRI intensities pre- and four hours post-IT contrast IT: Intrathecal

Location	Average change in intensity
White matter	98.2
Grey matter	224.5
Left white matter	97
Left grey matter	269.3
Right white matter	99.3
Right grey matter	179.7
Left hemisphere	183.2
Right hemisphere	139.5

**Figure 3:**
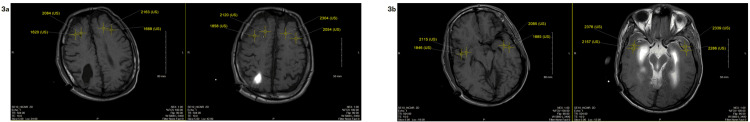
Patient 3 MRI intensities pre- and four hours post-IT contrast In each pair of images, the left image is pre-IT contrast, and the right image is four hours post-IT contrast. 3a: Frontal lobe; 3b: Temporal lobe IT: Intrathecal

Figure 3 illustrates the MRI intensities measured at each location. 

There was a higher average increase in intensity in grey matter when compared to white matter. Furthermore, there was also a higher average increase in intensity in the left side of the brain when compared to the right side of the brain.

The patient’s head CT scan demonstrated partial agenesis of the corpus callosum with less brain parenchyma on the right side. Having less functioning brain tissue on the patient’s right side of the brain may explain the higher increase in intensity in the left side of the brain because there is higher functioning brain tissue on the left side and higher uptake. 

Patient 4

A 67-year-old male patient presented with a chief complaint of left-sided weakness with an initial GCS13 (M6V4E3). Head CT demonstrated right temporal intraparenchymal hemorrhage with intraventricular hemorrhage and signs of hydrocephalus. Head CTA demonstrated a ruptured 4-millimeter aneurysm arising from the right posterior communicating artery and an unruptured eight-millimeter aneurysm from the right middle cerebral artery bifurcation. A right-sided EVD was placed for CSF diversion. A DSA was subsequently performed, and the patient underwent successful coil embolization of the ruptured aneurysm.

MRI brain scans were obtained pre-IT contrast, 12 hours post-IT contrast, and 36 hours post-IT contrast with GCS scores of 13 (M6V4E3), 10T (M6VTE3), and 10T (M6VTE3) at those respective timepoints. The pre-IT contrast MRI brain scan was performed 121 hours after the CTA and before the DSA. The 12-hour post-IT contrast MRI brain scan was performed 210 hours after the CTA and 63 hours after the DSA. The 36-hour post-IT contrast MRI brain scan was performed 234 hours after the CTA and 87 hours after the DSA.

Tables 8-11 display the MRI intensities of patient 4's brain parenchymal regions and lymph nodes pre-, 12 hours post-, and 36 hours post-IT contrast.

**Table 8:** Patient 4 brain parenchyma MRI intensities pre- and 12 hours post-IT contrast IT: Intrathecal; TR: Repetition time

Location	Pre-IT contrast intensity (TR 504)	12 hours post-IT contrast intensity (TR 546)	Change in intensity (pre- and 12 hours post-)	% change
Left frontal lobe (white matter)	1744	1282	-462	-26.5
Left frontal lobe (grey matter)	1463	1202	-261	-17.8
Right frontal lobe (white matter)	1798	1334	-464	-25.8
Right frontal lobe (grey matter)	1506	1106	-400	-26.6
Left temporal lobe (white matter)	2014	1383	-631	-31.3
Left temporal lobe (grey matter)	1856	1053	-803	-43.3
Right temporal lobe (white matter)	1720	1160	-560	-32.6
Right temporal lobe (grey matter)	1863	1035	-828	-44.4
Left parietal lobe (white matter)	1834	1367	-467	-25.5
Left parietal lobe (grey matter)	1567	1338	-229	-14.6
Right parietal lobe (white matter)	1783	1384	-399	-22.4
Right parietal lobe (grey matter)	1671	1200	-471	-28.2
Central pons (ventral to cerebral aqueduct)	2007	1497	-510	-25.4
Central medulla (ventral to fourth ventricle)	1961	1507	-454	-23.2
Left cerebellum (white matter)	2087	1320	-767	-36.8
Right cerebellum (white matter)	2013	1295	-718	-35.7

**Table 9:** Patient 4 brain parenchyma MRI intensities pre- and 36 hours post-IT contrast IT: Intrathecal; TR: Repetition time

Location	Pre-IT contrast intensity (TR 504)	36 hours post-IT contrast intensity (TR 525)	Change in intensity (pre- and 36 hours post-)	% change
Left frontal lobe (white matter)	1744	2457	713	40.9
Left frontal lobe (grey matter)	1463	2263	800	54.7
Right frontal lobe (white matter)	1798	2689	891	49.6
Right frontal lobe (grey matter)	1506	2273	767	50.9
Left temporal lobe (white matter)	2014	2846	832	41.3
Left temporal lobe (grey matter)	1856	2595	739	39.8
Right temporal lobe (white matter)	1720	3062	1342	78.0
Right temporal lobe (grey matter)	1863	2760	897	48.1
Left parietal lobe (white matter)	1834	2833	999	54.5
Left parietal lobe (grey matter)	1567	2672	1105	70.5
Right parietal lobe (white matter)	1783	3080	1297	72.7
Right parietal lobe (grey matter)	1671	2553	882	52.8
Central pons (ventral to cerebral aqueduct)	2007	3185	1178	58.7
Central medulla (ventral to fourth ventricle)	1961	3502	1541	78.6
Left cerebellum (white matter)	2087	3122	1035	49.6
Right cerebellum (white matter)	2013	3466	1453	72.2

**Table 10:** Patient 4 lymph node MRI intensities pre- and 12 hours post-IT contrast IT: Intrathecal; TR: Repetition time

Location	Pre-IT contrast intensity (TR 504)	12 hours post-IT contrast intensity (TR 546)	Change in intensity (pre- and 12 hours post-)	% change
Left retropharyngeal	1986	1086	-900	-45.3
Right retropharyngeal	1857	1005	-852	-45.9
Left posterior cervical	2305	1011	-1294	-56.1
Right posterior cervical	2516	1339	-1177	-46.8

**Table 11:** Patient 4 lymph node MRI intensities pre- and 36 hours post-IT contrast IT: Intrathecal; TR: Repetition time

Location	Pre-IT contrast intensity (TR 504)	36 hours post-IT contrast intensity (TR 525)	Change in intensity (pre- and 36 hours post-)	% change
Left retropharyngeal	1986	2045	59	3.0
Right retropharyngeal	1857	1997	140	7.5
Left posterior cervical	2305	2553	248	10.8
Right posterior cervical	2516	2547	31	1.2

Tables 12-13 display the average change in MRI intensities.

**Table 12:** Patient 4 average change in MRI intensities pre- and 12 hours post-IT contrast IT: Intrathecal

Location	Average change in intensity (pre- and 12 hours post-)
White matter	-497.2
Grey matter	-498.7
Left white matter	-520
Left grey matter	-431
Right white matter	-474.3
Right grey matter	-566.3
Left hemisphere	-475.5
Right hemisphere	-520.3

**Table 13:** Patient 4 average change in MRI intensities pre- and 36 hours post-IT contrast IT: Intrathecal

Location	Average change in intensity (pre- and 36 hours post-)
White matter	1012.3
Grey matter	865
Left white matter	848
Left grey matter	881.3
Right white matter	1176.7
Right grey matter	848.7
Left hemisphere	864.7
Right hemisphere	1012.7

**Figure 4:**
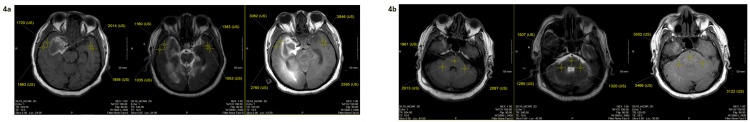
Patient 4 MRI intensities pre-, 12 hours post-, and 36 hours post-IT contrast In each triplet of images, the left image is pre-IT contrast, the middle image is 12 hours post-IT contrast, and the right image is 36 hours post-IT contrast. 4a: Temporal lobe; 4b: Medulla and cerebellum IT: Intrathecal

Figure 4 illustrates the MRI intensities measured at each location.

There was a global (both brain parenchyma and lymph nodes) decrease in intensity in the first MRI scan, 12 hours post-IT contrast. The patient’s clinical status also changed during that time point. He developed worsening altered mental status (GCS10T) and required intubation for airway protection. The deterioration in clinical status and decline in the patient’s GCS correlate with an underlying worsening intracranial process in this patient, especially one that would cause decreased glymphatic flow.

At the 36-hour post-IT contrast time point, the patient’s clinical status slowly improved, but he remained a GCS10T. By this time, the intensities (both brain parenchyma and lymph nodes) globally increased in all areas of the brain.

Patient 5

A 57-year-old male patient presented with a chief complaint of headaches with initial GCS14 (M6V4E4). Head CT demonstrated perimesencephalic subarachnoid hemorrhage with signs of hydrocephalus. A right-sided EVD was placed for CSF diversion. A DSA was subsequently performed and did not demonstrate any aneurysms, vascular malformations, or other vascular anomalies.

MRI brain scans were obtained pre-IT contrast and four hours-post IT contrast with GCS scores of 15 (M6V5E4) at both timepoints. The pre-IT contrast MRI brain scan was performed 119 hours after the CTA and 105 hours after the DSA. The post-IT contrast MRI brain scan was performed 184 hours after the CTA and 170 hours after the DSA.

Tables 14-15 display the MRI intensities of patient 5's brain parenchymal regions and lymph nodes pre- and four hours post-IT contrast.

**Table 14:** Patient 5 brain parenchyma MRI intensities pre- and four hours post-IT contrast IT: Intrathecal; TR: Repetition time

Location	Pre-IT contrast intensity (TR 546)	Four hours post-IT contrast intensity (TR 546)	Change in intensity	% change
Left frontal lobe (white matter)	1817	1682	-135	-7.4
Left frontal lobe (grey matter)	1563	1382	-181	-11.6
Right frontal lobe (white matter)	1828	1639	-189	-10.3
Right frontal lobe (grey matter)	1534	1387	-147	-9.6
Left temporal lobe (white matter)	2292	1936	-356	-15.5
Left temporal lobe (grey matter)	2039	1610	-429	-21.0
Right temporal lobe (white matter)	2035	1930	-105	-5.2
Right temporal lobe (grey matter)	1999	1577	-422	-21.1
Left parietal lobe (white matter)	1791	1622	-169	-9.4
Left parietal lobe (grey matter)	1329	1478	149	11.2
Right parietal lobe (white matter)	1732	1780	48	2.8
Right parietal lobe (grey matter)	1391	1496	105	7.5
Central pons (ventral to cerebral aqueduct)	1881	1747	-134	-7.1
Central medulla (ventral to fourth ventricle)	2047	1762	-285	-13.9
Left cerebellum (white matter)	1779	1751	-28	-1.6
Right cerebellum (white matter)	1879	1785	-94	-5.0

**Table 15:** Patient 5 lymph node MRI intensities pre- and four hours post-IT contrast IT: Intrathecal; TR: Repetition time

Location	Pre-IT contrast intensity (TR 546)	Four hours post-IT contrast intensity (TR 546)	Change in intensity	% change
Left retropharyngeal	2172	2209	37	1.7
Right retropharyngeal	2260	2291	31	1.4
Left posterior cervical	1821	1973	152	8.3
Right posterior cervical	1921	1969	48	2.5

Table 16 displays the average change in MRI intensities.

**Table 16:** Patient 5 average change in MRI intensities pre- and four hours post-IT contrast IT: Intrathecal; TR: Repetition time

Location	Average change in intensity
White matter	-151
Grey matter	-154.2
Left white matter	-220
Left grey matter	-153.7
Right white matter	-82
Right grey matter	-154.7
Left hemisphere	-186.8
Right hemisphere	-118.3

**Figure 5:**
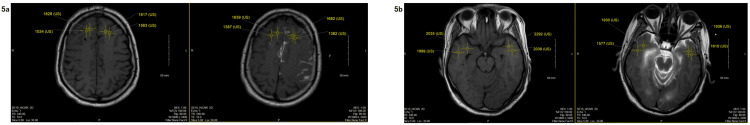
Patient 5 MRI intensities pre- and four hours post-IT contrast In each pair of images, the left image is pre-IT contrast and the right image is four hours post-IT contrast. 5a: Frontal lobe; 5b: Temporal lobe IT: Intrathecal

Figure 5 illustrates the MRI intensities measured at each location.

There was a global decrease in intensity in both hemispheres. In patients with perimesencephalic subarachnoid hemorrhage, the buildup of blood products in the arachnoid granulations impairs their ability to clear glymphatic fluid and metabolic byproducts. This would result in less clearance of the contrast out of the ventricular system into the brain parenchyma and may explain why there was no increase in intensity. The unusual decrease in intensity is explained further in this description due to increased first-pass concentrations or timing relationship to CTA and DSA. There were higher intensities in the right compared to the left, again suggesting that the EVD drains both ventricular CSF and facilitates drainage of glymphatic fluid.

There were relatively greater changes in the intensities of the left retropharyngeal and posterior cervical lymph nodes when compared to those on the right side. This is different than the decrease seen in brain parenchyma. The asymmetry seen could be due to dysfunction of the glymphatic system globally, causing some fluid pathways to be slower than others.

Patient 6

A 35-year-old male presented with a chief complaint of altered mental status and an initial GCS10T (M5VTE4). Head CT demonstrated diffuse subarachnoid hemorrhage with signs of hydrocephalus. The head CTA demonstrated a ruptured three-millimeter aneurysm arising from the anterior communicating artery. A right-sided EVD was placed for CSF diversion. A DSA was subsequently performed, and the patient underwent successful coil embolization of the ruptured aneurysm. The patient later developed an elevated ICP that was refractory to medical management and underwent a bifrontal craniectomy.

MRI brain scans were obtained pre-IT contrast and four hours-post IT contrast with GCS scores of 10T (M5VTE4) at both timepoints. The pre-IT contrast MRI brain scan was performed 162 hours after the CTA, 139 hours after the DSA, and 70 hours after the craniectomy. The post-IT contrast MRI brain scan was performed 388 hours after the CTA, 365 hours after the DSA, and 296 hours after the craniectomy.

Tables 17-18 display the MRI intensities of patient 6's brain parenchymal regions and lymph nodes pre- and four hours post-IT contrast.

**Table 17:** Patient 6 brain parenchyma MRI intensities pre- and four hours post-IT contrast IT: Intrathecal; TR: Repetition time

Location	Pre-IT contrast intensity (TR 546)	Four hours post-IT contrast intensity (TR 567)	Change in intensity	% change
Left frontal lobe (white matter)	1894	2145	251	13.3
Left frontal lobe (grey matter)	1656	1964	308	18.6
Right frontal lobe (white matter)	1802	1991	189	10.5
Right frontal lobe (grey matter)	1548	1787	239	15.4
Left temporal lobe (white matter)	2028	2410	382	18.8
Left temporal lobe (grey matter)	1765	2128	363	20.6
Right temporal lobe (white matter)	2027	2531	504	24.9
Right temporal lobe (grey matter)	1742	2273	531	30.5
Left parietal lobe (white matter)	1869	2113	244	13.1
Left parietal lobe (grey matter)	1637	1839	202	12.3
Right parietal lobe (white matter)	1899	2348	449	23.6
Right parietal lobe (grey matter)	1707	2027	320	18.7
Central pons (ventral to cerebral aqueduct)	2142	2515	373	17.4
Central medulla (ventral to fourth ventricle)	1715	2450	735	42.9
Left cerebellum (white matter)	1795	2397	602	33.5
Right cerebellum (white matter)	1761	2522	761	43.2

**Table 18:** Patient 6 lymph node MRI intensities pre- and four hours post-IT contrast IT: Intrathecal; TR: Repetition time

Location	Pre-IT contrast intensity (TR 546)	Four hours post-IT contrast intensity (TR 567)	Change in intensity	% change
Left retropharyngeal	1812	1825	13	0.7
Right retropharyngeal	1431	1473	42	2.9
Left posterior cervical	1514	1690	176	11.6
Right posterior cervical	1621	1765	144	8.9

Table 19 displays the average change in MRI intensities.

**Table 19:** Patient 6 average change in MRI intensities pre- and four hours post-IT contrast IT: Intrathecal

Location	Average change in intensity
White watter	336.5
Grey matter	327.2
Left white matter	292.3
Left grey matter	291
Right white matter	380.7
Right grey matter	363.3
Left hemisphere	291.7
Right hemisphere	372

**Figure 6:**
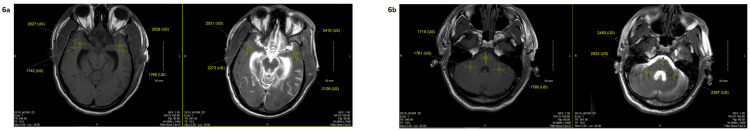
Patient 6 MRI intensities pre- and four hours post-IT contrast In each pair of images, the left image is pre-IT contrast and the right image is four hours post-IT contrast. 6a: Temporal lobe; 6b: Medulla and cerebellum IT: Intrathecal

Figure 6 illustrates the MRI intensities measured at each location.

There was a similar average increase in intensity in both white and grey matter. Furthermore, there was also a higher average increase in intensity in the right side of the brain with the EVD when compared to the left side of the brain. Patients with diffuse subarachnoid hemorrhage have arachnoid granulation dysfunction, resulting in impaired ability to clear CSF and metabolic byproducts. Since the damage is at the molecular and cellular level, it can be hard to see at a macroscopic level on an MRI scan. However, this patient likely had arachnoid granulation dysfunction affecting the left hemisphere more because there were asymmetric increases in intensity with higher increases in the right hemisphere. There were higher intensities in the right compared to the left, suggesting that the EVD drains both ventricular CSF and facilitates the drainage of glymphatic fluid.

There were relatively greater changes in intensities in the posterior cervical lymph nodes bilaterally compared to those of the retropharyngeal lymph nodes. This suggests the fluid pathways leading to the posterior cervical lymph nodes are different from retropharyngeal lymph nodes, causing them to receive the IT contrast first.

## Discussion

High ICP frequently occurs in cases of severe head injuries and intracranial tumors, infarction, and hemorrhage. Substantial evidence indicates a direct correlation between elevated ICP in severely brain-injured patients and an impaired neurological outcome, often attributed to reduced cerebral perfusion pressures (CPPs) and reduced cerebral blood flow [18]. In individuals with severe TBI and heightened ICP, CSF is externally drained using EVDs. While the arachnoid granulations play a primary role in absorbing the majority of CSF, the waste products also travel through the VRS with the glymphatic system, serving as another significant pathway for ISF outflow [19]. It connects the central and peripheral lymphatic systems within the meninges, where fluid is absorbed. CSF absorption rate correlates with visualization of the IT contrast in this study. The glymphatic system, involving astrocytes, plays a more substantial role than previously thought in clearing cerebral waste and reducing interstitial brain edema. This mechanism utilizes CSF pulsations along perivascular spaces, connecting deep cerebral blood vessels, leptomeningeal sheaths, and AQP-4 water channels, through the glymphatic system, which then ultimately drains into the cervical lymphatic system [20]. This study demonstrates that the glymphatic system functions at different rates in different tissues. It also demonstrates that fluid through the glymphatic system is enhanced with EVD CSF drainage, either through decreasing local ICP or another mechanism.

The primary brain injury from intraparenchymal hemorrhages leads to secondary brain injuries such as severe edema, ischemia, and dangerously elevated ICP. When the disease is due to underlying arteriosclerosis, there are diminished arterial pulsations resulting in decreased CSF fluid traveling along the VRS. AQP-4 channel function upregulation and fluid uptake through the brain substance are needed for convective bulk glymphatic fluid movement through the intraparenchymal space and cells, which is impaired by intracranial hemorrhage and other diseases. The extent of these secondary injuries is often demonstrated on head CT or MRI brain scans with imaging findings of effacement of sulci and gyri, mass effect on nearby brain structures, and herniation of brain structures onto the brainstem. Gadoterate meglumine (Dotarem) serves as a CSF and glymphatic tracer and is visualized on MRI imaging when brain structures appear brighter or the intensity value increases. Asymmetric changes in intensity values between the right hemisphere and left hemisphere reflect different fluid flow rates, signaling underlying structural changes that may be obstructing glymphatic outflow. It has been shown in this study to also be affected by EVD drainage. Less contrast enhancement intensity after IT contrast administration suggests less CSF influx into the peri-arterial space, less flow through the glymphatic pathway, and therefore less efflux from the peri-venous space into the cervical lymph nodes. The AQP-4 channels may have also become dysfunctional or less upregulated after primary and secondary brain injuries. As demonstrated in this study, early after intracranial hemorrhage and ischemic stroke, there is a hypermetabolism of fluid into the glymphatic system with an increase in AQP-4 channels through upregulation of mRNA expression, suggesting the need to improve flow to clear up the accumulation of fluid, metabolites, and byproducts of primary and secondary brain injury and negative IT MRI contrast enhancement due to the first pass phenomenon. It is also demonstrated that hypermetabolism may occur throughout the glymphatic system with increased clearance into the cervical lymph nodes.

There are several rare scenarios that will cause a negative enhancement with MRI gadolinium-based contrast enhancement, as seen in this study. There is a paradoxical decrease in signal intensity (darkening) on T1-weighted images, even when maintaining the same TE, TR, and window levels [21]. This phenomenon is almost exclusively driven by extreme, localized concentrations of the contrast agent, which shift the magnetic property effect from T1-shortening (bright) to T2/T2-shortening (dark). High-concentration "T2 Susceptibility" affects areas with very high concentrations of gadolinium, and the T2-shortening effect dominates over the T1-shortening effect. This results in signal loss (darkening) rather than signal gain (brightening). This is most commonly seen in the "first pass" of contrast through vessels, or in instances of inadvertent high-concentration pooling, intra-articular or intravascular injection, when high-concentration contrast is injected directly into a joint (arthrogram) or vein, the high local concentration can lead to signal drop-out rather than enhancement, appearing as a dark area on the image. With a shorter TR (with 504 being the lowest in this series), the overall image signal intensity decreases because tissues have less time to recover, leading to less magnetization being available for the next pulse, while T1 contrast signal intensity increases. A longer TR (with 609 being the longest in this series) increases overall signal intensity and decreases signal intensity on T1 contrast; water and CSF appear darker, while fat appears brighter.

Variation in repetition time (TR) across imaging timepoints represents a potential confounder, as T1-weighted signal intensity is partially dependent on TR. In this series, TR ranged from 504 to 609 ms. However, several observations suggest that TR variation alone does not account for the regional and temporal differences in signal intensity observed. First, changes in signal were spatially heterogeneous and often asymmetric, which would not be expected from a global acquisition parameter such as TR. Second, regions presumed to be unaffected by glymphatic flow alterations (e.g., contralateral white matter and cerebellum) did not demonstrate proportional changes in signal intensity across timepoints, suggesting relative internal consistency within each scan. Finally, the magnitude and distribution of signal changes correlated more closely with clinical status and underlying pathology than with acquisition parameters. While normalization to a reference tissue could further mitigate this limitation, the internal comparisons within each scan support that the observed signal differences primarily reflect biological variation in glymphatic flow rather than technical variability.

Patient 2 had a negative contrast intensity at 12 hours only in the left posterior cervical node, possibly due to increased drainage from the hypermetabolism through the glymphatic system into the lymph nodes, possibly corresponding to the improved GCS after the craniotomy. The other possibility is that it may be due to the time relationship to DSA, as iodinated contrast agents (e.g., iopamidol, iopromide) can cause shortening of T1, T2, and T2 relaxation times, altering the signal on various MRI sequences. The GCS improved prior to the post-IT contrast MRI. The pre-IT contrast MRI brain scan was performed 26 hours after the CTA and before the DSA and craniotomy. The post-IT contrast MRI brain scan was performed 297 hours after the CTA, 240 hours after the first DSA, 152 hours after the second DSA, and 129 hours after the craniotomy and therefore is unlikely due to the additional iodinated contrast material. There were no changes in the remainder of the quantified areas. The initial bleed was in the left temporal lobe. The TR was 546 initially, and TR 609 at 12 hours.

Patient 3 had a negative contrast intensity at four hours in the left parietal white and grey areas, as well as the medulla and right cerebellum. The initial injury was hydrocephalus. The TR was 504 initially and at four hours. There was a history of right-brain damage from birth. The right-sided EVD could have decreased intrinsic brain pressure and permitted the functioning brain on the left and posterior fossa to increase metabolism over the pre-contrast time scan, resulting in the first-pass phenomenon, high-contrast load pooling.

Patient 4 had a negative contrast intensity at 12 hours in all areas, including cervical lymph nodes, when the patient’s mentation decreased. The GCS decreased prior to the MRI. The possible reasons for this decrease include either the opening of the BBB associated with the decrease in GCS, the first-pass phenomenon causing shortening of T1, T2, and T2 relaxation times (which can alter the signal on various MRI sequences), or it may be due to another unexplained process. The 12-hour post-IT contrast MRI brain scan was performed 210 hours after the CTA and 63 hours after the DSA, and therefore, the additional iodinated contrast agents as the cause is unlikely. The negative contrast intensity recovered at 36 hours. The initial bleed was in the right temporal lobe, and a right-sided EVD was placed. The TR was 504 initially, increased to 546 at 12 hours, and decreased to 525 at 36 hours. 

While no procedure-related complications were observed immediately following intrathecal contrast administration in this series, the clinical deterioration observed in patient 4 warrants careful interpretation. Although the temporal association raises concern, causality between intrathecal gadolinium administration and neurological decline cannot be established from this single case, particularly given the presence of multiple confounding factors, including underlying hemorrhage, elevated intracranial pressure, EVD dependence, and evolving secondary injury. Prior human studies evaluating intrathecal gadolinium-based contrast agents, including prospective safety cohorts using intrathecal gadobutrol in 100 patients, have reported no serious adverse events and overall tolerability of doses up to 0.5 mmol, though minor adverse effects such as nausea have been reported [8,22]**.** Additional human cohorts investigating glymphatic imaging with intrathecal gadobutrol in large patient series have similarly demonstrated clinical feasibility without major safety signals [23,24]**.** Therefore, while existing evidence supports the relative safety of low-dose intrathecal gadolinium administration in humans, our findings do not establish definitive safety, nor do they exclude the possibility of idiosyncratic or context-dependent effects in critically ill patients. The observed decline in patient 4 may represent either impaired glymphatic transport related to worsening intracranial pathology or a transient susceptibility effect related to contrast kinetics rather than a direct toxic effect of the contrast agent itself. As such, we interpret these results as hypothesis-generating and emphasize the need for larger controlled studies to more definitively characterize safety, causality, and dose-response relationships.

Patient 5 had a negative contrast intensity at four hours in all areas, except the parietal lobes and cervical lymph nodes, without a change in mentation. The initial bleed was mesencephalic SAH, and a right-sided EVD was placed. The TR was 546 at both times. The pre-IT contrast MRI brain scan was performed 119 hours after the CTA and 105 hours after the DSA. The four-hour post-IT contrast MRI brain scan was performed 184 hours after the CTA and 170 hours after the DSA. Similar to patient 4, it is unlikely due to the additional iodinated contrast agents. The negative contrast enhancement was holocranial and not associated with a localized brain injury, a change in GCS from pre-IT contrast, or localized to the right-sided EVD. Therefore, the negative contrast enhancement at four hours post-injection is indeterminate, possibly related to the delayed effects of the EVD increasing neuronal activity with upregulation of AQP-4 channels, first-pass phenomenon with high contrast load pooling (but without clearing of the fluid and solute through the glymphatic system), or another unknown reason. It does not appear that the patients in this study were affected by a change in the TR on MRI scanning. The TE remained the same at 10, and the calculated intensities were with window levels of W5800 and L3400.

Paradoxical signal reduction following intrathecal gadolinium administration was observed in patients 4 and 5. While several mechanisms may contribute to this phenomenon, the most likely explanation is transient T2/T2* susceptibility effects from locally elevated contrast concentration during early distribution (“first-pass” phenomenon), resulting in signal loss on T1-weighted imaging. This mechanism is supported by the timing of imaging relative to contrast administration and the subsequent normalization of signal intensity at later timepoints, particularly in patient 4. Alternative explanations include residual effects of prior iodinated contrast exposure or blood-brain barrier disruption; however, the temporal separation from angiographic studies and lack of consistent global signal changes make these less likely primary drivers.

Distinguishing between true glymphatic dysfunction and susceptibility-related signal effects remains challenging. In future studies, this may be addressed through normalization to internal reference tissues, use of multi-sequence imaging (including T2-weighted or susceptibility-weighted imaging), and correlation with quantitative flow metrics or serial timepoint analysis. Taken together, the findings most strongly support a dominant contribution from contrast concentration-related susceptibility effects, with potential superimposed biological impairment of glymphatic flow in clinically deteriorating patients.

In the normal physiological state, IT contrast enters the CSF space, then VRS, then the astrocytic foot process, and AQP-4 channels. In the pathological disease state, there is a disrupted BBB that is more permeable to fluids and other substances. If previously given iodinated contrast agents during angiography, such as in patients 4 and 5, there may be temporary damage to the BBB from hyperosmolar substances, causing endothelial cells to shrink and widen tight junctions, allowing fluid and contrast to leak into brain tissue, which then leads to cerebral edema.

This is the first study to deliver IT gadoterate meglumine (Dotarem) via an EVD to study and evaluate the glymphatic system and flow. No patients developed any adverse events from the IT contrast administration. Many of the patients experienced asymmetric increases in MRI image intensity that correlated with their localized lack of intracranial pathology that would obstruct glymphatic outflow. This study demonstrated reduced glymphatic flow (decreased MRI intensity and less uptake of IT contrast) in patients with a decline in GCS or focal lesions such as unilateral intracranial hemorrhage. Truncated glymphatic flow leads to the buildup of interstitial edema, which can ultimately lead to elevated ICP and cause life-threatening hydrocephalus in patients. Treatment involves CSF and glymphatic diversion, usually in the form of emergent EVD placement.

The observed association between decreased GCS and reduced glymphatic signal intensity is consistent with established experimental data demonstrating disruption of perivascular water transport following brain injury. In particular, subarachnoid hemorrhage and other acute brain insults have been shown to induce depolarization and mislocalization of AQP-4 channels away from astrocytic endfeet, impairing perivascular CSF-interstitial fluid exchange. Experimental models of glymphatic function, including murine studies of subarachnoid hemorrhage, have demonstrated that loss of perivascular AQP-4 polarization significantly reduces glymphatic influx and clearance [2,25]. This mechanistic framework provides a biologically plausible explanation for the reduced contrast propagation observed in patients with neurological decline, suggesting that impaired glymphatic transport may contribute to worsening cerebral edema and decreased neurological function.

This study provides a framework for evaluating glymphatic function in vivo using intrathecal contrast administration via EVD. This methodology may enable future investigation of therapeutic strategies aimed at modulating glymphatic flow, including interventions that influence intracranial pressure, vascular pulsatility, and lymphatic drainage. Such approaches may include both established medical and surgical therapies as well as adjunctive techniques designed to enhance cerebrospinal fluid dynamics. Osteopathic manipulative medicine (OMM) or osteopathic manipulative treatment (OMT) has demonstrated efficacy in reducing ICP, possibly through its effects on the glymphatic system. Thoracic pump and pedal pump techniques decrease ICP and increase CPP [26], possibly through the rhythmic expansion and contraction of the cerebral arteries and through increased venous return driving the convective bulk flow. Both interventions target the patient’s lymphatic system to improve overall fluid balance and prevent the buildup of metabolic waste and edema. CV4 is another technique that was studied and resulted in clinical and radiographic improvement in a patient with normal pressure hydrocephalus. The technique enhances the patient’s cranial rhythmic impulse and synchronizes brain activity bilaterally as measured on electromagnetic field (EMF) waveforms, signaling optimization of CSF flow into and through the glymphatic system [27,28]. This brain EMF was also increased with EMF stimulation [29,30]. Additional techniques that further improve the patient’s circulatory system target venous sinus drainage. Another study investigated the role of 51-minute sessions of various osteopathic techniques targeting the central and peripheral glymphatic systems in TBI patients with elevated ICP. The results demonstrated improvement in the patients’ ICP, optic nerve sheath diameter measurements, and neurologic pupil index scores, suggesting that OMT is effective in improving CSF flow [31]. The common theme underlying the thoracic pump, pedal pump, CV4, and other venous sinus drainage techniques is to improve CSF influx in the peri-arterial space and out through the perivenous space, which can help restore normal glymphatic drainage. It would be helpful to identify IT injection of GBCAs in the cervical lymph nodes at certain timepoints after multiple interventions, such as OMT, to further understand enhancement of the glymphatic system.

This study demonstrates that IT administration of GBCAs via EVD has relative safety, but definitive safety cannot be concluded. Additionally, IT GBCA can be visualized on MRI brain scan imaging at various time points, outlining the different functions of different parts of the glymphatic system in different brain tissues. Additional studies can further investigate the effects of increasing CSF and glymphatic flow through the ventricles, VRS, and brain parenchyma using different modalities, such as OMT, by performing techniques before and after IT contrast administration via EVD. The optimal timing of serial MRI brain scans needs to be studied, but considerations include pre-IT contrast followed by four hours-post IT contrast, 12 hours-post IT contrast, and 24 hours-post IT contrast [7]. Further studies at four hours post IT contrast may answer the question of whether it is too early for a diagnosis due to the first-pass phenomenon.

Limitations

This study involves a limited number of patients receiving serial MRI scans of the brain and neck. Further studies should investigate the optimal timing of serial MRI brain scan acquisition to characterize IT contrast uptake into the glymphatic system and processes that change glymphatic enhancement and clearance into the cervical lymph nodes.

## Conclusions

The glymphatic system is a critical pathway for cerebrospinal fluid-interstitial fluid exchange and plays an important role in maintaining intracranial homeostasis. In acute intracranial conditions such as hemorrhage, traumatic brain injury, and hydrocephalus, disruption of glymphatic function may contribute to cerebral edema, impaired waste clearance, and elevated intracranial pressure. MRI-based glymphatic imaging suggests that brain injury may increase water influx into glymphatic pathways, potentially through upregulation or redistribution of AQP-4 water channels. However, when downstream clearance is impaired, this influx may lead to stagnation of fluid, accumulation of metabolic byproducts, interstitial fluid buildup, and worsening edema. In this series, intrathecal gadolinium-based contrast administered through an external ventricular drain allowed visualization of glymphatic dynamics in patients with acute intracranial pathology. Imaging demonstrated heterogeneous, region-specific tracer distribution patterns that appeared to correlate with underlying disease processes and clinical status.

These findings likely reflect a combination of altered glymphatic flow, AQP-4-mediated water transport abnormalities, contrast kinetics, susceptibility effects, and disease-related physiologic changes rather than a single mechanism. Although no immediate procedural complications were observed, the clinical deterioration seen in one patient underscores the need for caution, as safety and causality cannot be determined from a small, heterogeneous cohort. Prior human studies have reported overall tolerability of low-dose intrathecal gadolinium, but critically ill patients with compromised blood-brain barrier integrity and evolving secondary injury may represent a particularly vulnerable subgroup. Taken together, this study supports the feasibility of intrathecal contrast-enhanced MRI for in vivo assessment of glymphatic function, while emphasizing that definitive conclusions regarding safety, causality, and clinical impact remain limited. Larger prospective studies with standardized dosing protocols and longitudinal monitoring are needed to clarify the role of glymphatic dysfunction in secondary brain injury and define the clinical utility and safety profile of intrathecal contrast-based imaging.
